# Seroprevalence of anti-SARS-CoV-2 IgG antibodies pre- and post-COVID-19 vaccination in staff members of Bandar Abbas Children’s Hospital

**DOI:** 10.1186/s12879-023-08863-z

**Published:** 2024-02-23

**Authors:** Marjan Tariverdi, Hossein Mohammadi, Farideh Hassanzadeh, Mohammad Tamaddondar

**Affiliations:** 1 Department of Pediatrics, Faculty of Medicine, Hormozgan University of Medical Sciences, Bandar Abbas, Iran; 2 Student Research Committee, Hormozgan University of Medical Sciences, Bandar Abbas, Iran; 3 Department of Pediatrics, Clinical Research Development Center of Children’s Hospital, Hormozgan University of Medical Science, Bandar Abbas, Iran; 4 Department of Nephrology and Internal Medicine, Shahid Mohammadi Hospital, Hormozgan University of Medical Sciences, Bandar Abbas, Iran

**Keywords:** COVID-19, Healthcare workers, Seroprevalence, Vaccination

## Abstract

**Background:**

Healthcare workers (HCWs) have a higher risk of contracting coronavirus disease 2019 (COVID-19) compared to the general population due to their frontline role and direct contact with the infected patients. Accordingly, they were among the first groups to receive vaccination against COVID-19. A higher risk of COVID-19 infection may also exist among hospital staff members other than HCWs. In this study, we assessed the seroprevalence of anti-severe acute respiratory syndrome coronavirus 2 (SARS-CoV-2) IgG pre- and post-COVID-19 vaccination in hospital staff members.

**Methods:**

This cross-sectional study included 228 staff members of Bandar Abbas Children’s Hospital, Bandar Abbas, Iran, who were recruited from 2020 to 2021. Staff members were vaccinated with vector and inactivated vaccines. Anti-SARS-CoV-2 spike protein IgG was measured in their blood samples pre- and post-COVID-19 vaccination.

**Results:**

Of the 228 hospital staff members evaluated in this study (mean age: 37.59 ± 8.70 years), 204 (89.5%) were female and 210 (92.1%) were HCWs. Only one staff member was not vaccinated, the rest received one dose (99.6%), and 224 (98.7%) two doses. Vector vaccines were administered to 71.4% of staff members and 72.9% of HCWs. Anti-SARS-CoV-2 IgG antibody was positive in 8.8% of staff members before vaccination, 9.3% after the first dose, and 50% after the second dose. The corresponding percentages were 9.5%, 9.5%, and 48.8% in HCWs. Being a HCW was not associated with the seroprevalence of anti-SARS-CoV-2 IgG after the second dose; however, multivariable binary logistic regression analysis revealed that the interval between two vaccine doses (adjusted odds ratio [aOR] = 0.595, 95% confidence interval [CI] 0.434; 0.816, P = 0.001) and age (aOR = 1.062, 95% CI 1.021; 1.105, P = 0.003) were associated with seroprevalence.

**Conclusions:**

After receiving a second dose of vector or inactive virus vaccines, our hospital’s staff members and HCWs had a seroprevalence of anti-SARS-CoV-2 IgG antibodies of around 50%. Seroprevalence increased with increasing age and shorter intervals between doses.

## Introduction

The coronavirus disease 2019 (COVID-19) outbreak initially emerged as a local epidemic in Wuhan, China in December of 2019. However, its swift spread resulted in a worldwide pandemic that affected nearly all nations and led to substantial fatalities [1, 2]. Healthcare professionals, commonly referred to as healthcare workers (HCWs), are the primary workforce responsible for providing clinical care. It is widely believed that they are at a greater risk of contracting the disease compared to the general population, given their frontline role in healthcare provision. In the event of infection, HCWs not only present a potential hazard to susceptible patients but also to their colleagues in the healthcare profession [3, 4]. Moreover, the infection’s morbidity and associated stress can result in the disturbance of patient care [5].

Vaccination is considered the most effective measure for preventing COVID-19 infection [6]. Currently, there are four main types of COVID-19 vaccines, namely mRNA vaccines (Pfizer-BioNTech, Moderna), vector vaccines (AstraZeneca, Sputnik V, Johnson & Johnson), protein subunit vaccines (Novavax), and inactivated virus vaccines (Sinopharm, Bharat, Barekat) [7].

Despite the initiation of vaccination for Iranian HCWs in March 2021, there exist a dearth of scholarly literature pertaining to the seroprevalence of anti-severe acute respiratory syndrome coronavirus 2 (SARS-CoV-2) antibodies in HCWs subsequent to vaccination. Moreover, hospital staff members other than HCWs may also be at higher risk of COVID-19 infection than the general population.The investigation of the impact of protective measures, such as vaccination, on the production of antibodies can be effectively carried out by studying HCWs. This population is particularly suitable for such research as they were among the first to receive vaccinations and are in direct contact with patients infected with SARS-CoV-2.

we aimed to investigate the seroprevalence after the second round of vaccination of administrative and non-administrative personnel in order to investigate the effect of the second round of vaccine injection on the IgG antibody level against SARS-CoV-2. We measured the seroprevalence of IgG antibody against SARS-CoV-2 in the personnel of Bandar Abbas Children’s Hospital to reduce the lack of information about the effectiveness of the vaccine in hospital personnel.

## Methods

### Trial design and oversight 

A descriptive-cross sectional study was conducted at the Children’s Hospital in Bandar Abbas (located in Hormozgan province in southern Iran) to investigate the seroprevalence of anti-SARS-CoV-2 antibodies among hospital staff. For this purpose, we obtained ethical approval from the ethics committee of the Hormozgan University of Medical Sciences. Hospital staff at the Children’s Hospital in Bandar Abbas who were employed between March 2020 and March 2021 were included in the study, and samples were sent to the hospital’s own laboratory center. Seroprevalence of IgG antibodies before and after vaccination was measured along with demographic information.

### Trial participants

All Healthcare workers (HCWs), service and administrative staffs (non- HCWs) that employed at the Children’s Hospital in Bandar Abbas between 2020 and 2021 were eligible to participate in the study. Each individual was reviewed after obtaining written consent to participate in the study. Totally, 228 personnel were investigated. General information including age, gender, occupation, history of contact with a COVID-19 patient in the family, underlying diseases, and history of COVID-19 vaccination was obtained using a checklist developed under the supervision of infectious disease experts at the Children’s Hospital. Written consent was obtained from each individual prior to their review. A venous blood sample was taken from each person to measure their IgG antibody titers against SARS-CoV-2.

The following variables were also evaluated and recorded for all subjects:

- Occupation (HCW, non-HCW);
- Workplace (COVID wards, non-COVID wards).
- Underlying diseases (chronic pulmonary disease, cardiovascular disease, hypertension, diabetes, immunosuppression);
- Vaccine type and number of doses;
- The interval between the two vaccine doses;
- History of previous COVID-19 infection.

### Trial procedures and outcomes

A 3 cc blood sample was taken from each individual according to clinical and laboratory standards. The samples were transferred to a laboratory located in the hospital using a specialized container with ice. The samples were kept in a refrigerator for a maximum of 48 h before measuring their IgG antibody titers using the enzyme-linked immunosorbent assay (ELISA) method with the Euroimmune kit. Prior to receiving the vaccine and two weeks after receiving the first and second doses, each individual was evaluated for IgG antibodies.

The interval between the two vaccine doses depended on the type of vaccine, which is why the hospital staff were followed up through the vaccination program. After receiving the first dose, they were contacted, and then two weeks later, samples were taken. When receiving the second dose, confirmation was obtained via telephone. Finally, all staff received their second dose by March 2021, and two weeks after second dose, their IgG were assessed.

### Statistical analysis

Sampling method was census and descriptive statistics methods were used. The data analysis was conducted using the SPSS software (version 26.0). The statistical measures of mean and standard deviation were employed to describe continuous variables. Categorical variables were described using frequencies and percentages. Binary logistic regression analysis was applied to determine the factors associated with the seroprevalence of anti-SARS-CoV-2 IgG antibody. Accordingly, crude odds ratio (cOR), adjusted OR (aOR), and 95% confidence intervals (Cis) were reported. Statistical significance was determined by considering P-values less than 0.05.

## Results

Of the 228 hospital staff members evaluated in this study (mean age: 37.59 ± 8.70 years), 204 (89.5%) were female and 24 (10.5%) were male. Table 1 represents the general characteristics of the study participants. The majority of staff members were HCWs (92.1%) and worked in non-COVID wards (88.2%).

**Table 1:** General characteristics of the hospital staff members

			mean (SD)	
Age			37.59 (8.70) years	
Weight			64.58 (12.66) kg	
Height			161.65 (12.50) cm	
BMI			25.90 (16.46) (kg/m^2^)	
			Number	Percentage
Sex				
	Male		24	10.5
	Female		204	89.5
Occupation				
HCW			210	92.1
Non-HCW			18	7.9
Workplace				
COVID wards			27	11.8
Non-COVID wards			201	88.2
Underlying disease				
Yes			11	4.8
No			217	95.2
Contact with infected family member				
Yes			113	49.6
No			115	50.4
COVID-19 infection history				
Yes			14	6.1
No			214	93.9
Vaccination				
Yes			227	99.6
No			1	0.4
Vaccination doses				
One dose			227	99.6
Two doses			224	98.7
Interval mean between two doses (SD)			1.60(1.11) months	

Abbreviations: COVID-19, coronavirus disease 2019; HCW, healthcare worker

Only one staff member was not vaccinated, the rest received one dose (99.6%), and 224 (98.7%) received two doses. Vector vaccines were administered to 162/227 staff members (71.4%) as the first dose and to 160/224 (71.4%) as the second dose. All the 210 HCWs received vaccination, but only 207 (98.6%) received the second dose. Vector vaccines were administered to 153/210 HCWs (72.9%) as the first dose and to 151/207 (72.9%) as the second dose.

**Fig. 1:**
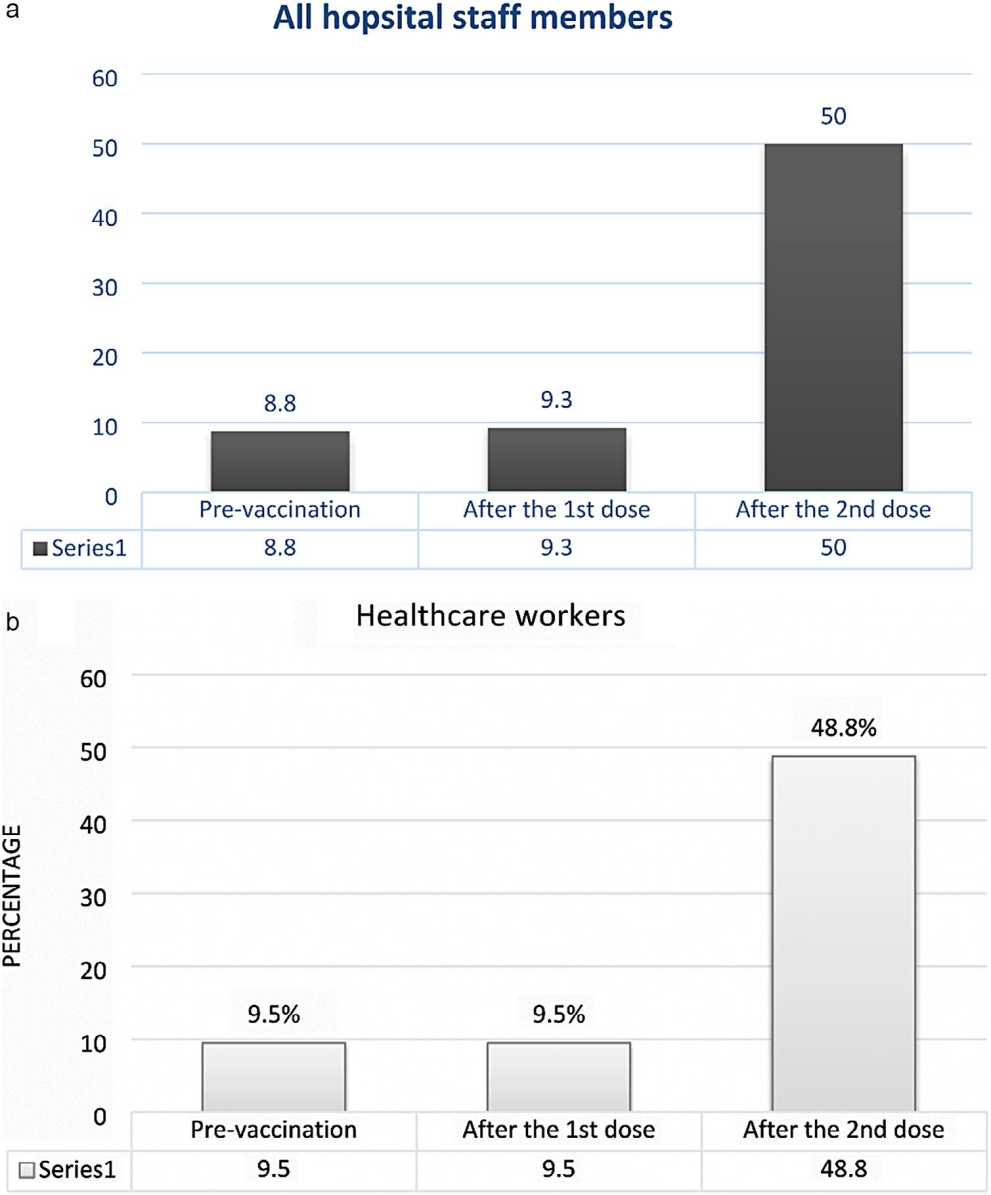
Seroprevalence of anti-SARS-CoV-2 IgG antibody in (**a**) hospital staff members; and (**b**) healthcare workers. This figure shows the positive rate of Anti-SARS-CoV-2 IgG antibody after the first dose of vaccine and the second dose of vaccine in two groups (health care and non-health care) of employees

The mean interval between the two vaccine doses was 1.60 ± 1.11 month. Anti-SARS-CoV-2 IgG antibody was positive in 8.8% (20/228) staff members before vaccination, in 9.3% (21/227) after the first dose, and in 50% (112/224) after the second dose (Fig. 1a). On the other hand, Anti-SARS-CoV-2 IgG antibody was positive in 9.5% (20/210) HCWs before vaccination, in 9.5% (20/210) after the first dose, and in 48.8% (101/207) after the second dose (Fig. 1b).

**Fig. 2:**
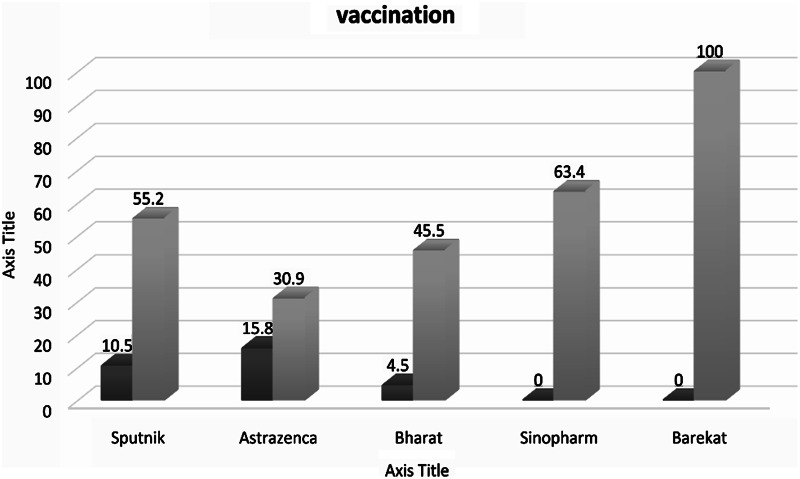
Seroprevalence after the first round and after the second round of vaccination

After the first dose, Seroconversion rates were highest with seroprevalence in AstraZeneca, while in the second stage, more seroprevalence was allocated to the Barekat (an Inactivated Iran-made vaccine that was available in the second dose of the vaccination program). Sinopharm shows a 63% increase in the second dose compared to the first dose. The difference between the seroconversion rates of the AstraZeneca vaccine between the first and second doses has significantly decreased, with the lowest amount of seroprevalence in the second dose. Bharat vaccine shows a tenfold growth in the second dose. Although Sputnik has grown compared to the first dose, it is less than the growth rate of Bharat vaccine. (Fig. 2).

Multivariable binary logistic regression analysis revealed that with every one-month increase in the interval between the two vaccine doses, the odds of positive anti-SARS-CoV-2 IgG (compared to negative IgG) decreased by almost 40% (aOR = 0.595, 95% CI 0.434; 0.816, p < 0.05). Also, every one-year increase in age increased the odds of positive anti-SARS-CoV-2 IgG by 6% (aOR = 1.062, 95% CI 1.021; 1.105, p < 0.05). However, being a HCW was not associated with the seroprevalence of anti-SARS-CoV-2 IgG (Table 2).

**Table 2:** Binary logistic regression analysis to determine the factors associated with the seroprevalence of SARS-CoV-2 IgG after the second dose of vaccine in hospital staff members

Independent variables	cOR (95% CI)	P-value	aOR (95% CI)	P-value
Age (years)	1.041 (1.008; 1.076)	p < 0.05	1.062 (1.021; 1.105)	p < 0.05
Sex				
Male	1.457 (0.618; 3.435)	p > 0.05	1.134 (0.346; 3.716)	p > 0.05
Female	1.000		1.000	
BMI (kg/m^2^)	0.998 (0.981; 1.015)	p > 0.05	0.997 (0.979; 1.014)	p > 0.05
Occupation				
HCW	0.520 (0.185; 1.458)	p > 0.05	0.501 (0.156; 1.604)	p > 0.05
Non-HCW	1.000		1.000	
Workplace				
COVID wards	1.530 (0.676; 3.464)	p > 0.05	2.774 (0.984; 7.823)	p > 0.05
Non-COVID wards	1.000		1.000	
Underlying disease	0.826 (0.244; 2.787)	p > 0.05	0.405 (0.094; 1.734)	p > 0.05
Contact with infected family member	0.542 (0.319; 0.921)	p < 0.05	0.568 (0.302; 1.069)	p > 0.05
Interval between two doses (months)	0.640 (0.490; 0.835)	p < 0.05	0.595 (0.434; 0.816)	p < 0.05
Vaccine type				
Vector	1.000		1.000	
Inactivated virus	1.553 (0.865; 2.788)	p > 0.05	1.090 (0.538; 2.210)	p > 0.05
History of COVID-19 infection	0.736 (0.247; 2.194)	p > 0.05	1.251 (0.369; 4.234)	p > 0.05

Abbreviations: aOR, adjusted odds ratio; BMI, body mass index; CI, confidence interval; cOR, crude odds ratio; COVID-19, coronavirus disease 2019; HCW, healthcare worker

## Discussion

We assessed the seroprevalence of anti-SARS-CoV-2 spike protein IgG antibodies among the staff members of a children’s hospital and found that pre-vaccination, seroprevalence was 8.8% in all staff members and 9.5% in HCWs. After receiving the first vaccine dose, seroprevalence increased to 9.3% in all staff members but remained unchanged in HCWs (9.5%). After the second vaccine dose, seroprevalence considerably increased to 50% in all staff members and 48.8% in HCWs.

Assaid et al. reported a seroprevalence of 65.9% by Euroimmun ELISA five months after the second dose of vector or inactivated virus vaccines in HCWs. The antibody response did not differ significantly between HCWs who received either vaccine type [8], which is consistent with our results, showing no relationship between vaccine type and seroprevalence by adjusted logistic regression analysis. The higher seroprevalence in Assaid et al.’s study can be justified by demographic and anthropometric differences and history of prior COVID-19 infection as none of the HCWs in Assaid et al.’s study had a history of COVID-19 infection [8]. A small group of our subjects were previously infected with COVID-19, but we found no association between such history and anti-SARS-CoV-2 IgG seroprevalence. Nonetheless, a single dose of the vaccine may be sufficient to induce an effective response in previously infected individuals, suggested by Gobbi et al. [9]

Interestingly, the seroprevalence of anti-SARS-CoV-2 after two doses of vector vaccines was 91.7% in HCWs of the study by Elangovan in India. They also observed a significant increase in antibody levels of HCWs who had a history of COVID-19 infection within six months prior to vaccination [10]. The much lower seroprevalence in our study might be due to almost one-third of subjects receiving inactivated virus vaccines, the interval between two doses of vaccines, as well as demographic differences and work settings. More importantly, the accuracy of measurements is always a matter of concern when evaluating laboratory parameters

Another explanation for the higher seroprevalence in Assaid et al.’s study [8] can be the time of antibody assessment. We evaluated anti-SARS-CoV-2 antibodies at least two weeks after the second vaccine dose while their measurements were done five months after the second dose. The two-week interval was chosen in our study because according to previous investigations, individuals vaccinated at least 14 days before antibody measurements were presumed to be seronegative [11]. However, Costa et al. reported higher antibody values with shorter time lapse around two to eight weeks between vaccination and serology [12]

Another finding of the present study was the positive correlation of age with anti-SARS-CoV-2 IgG seroprevalence after the second dose of vaccination as every one-year increase in age increased the odds of positive anti-SARS-CoV-2 IgG by 6%. Yet, the oldest subject in our study was 60 years old. Contrary to our findings, by studying antibody responses in 212,102 individuals, Ward et al. showed a decrease in antibody response with age, but this reduction was most prominent at ages 75 years and above [13]. On the other hand, we found no association between sex and seroprevalence. Conversely, Costa et al. reported lower serological levels in males [12]. A lower antibody response to mRNA vaccines has been demonstrated in men compared to women in other studies [14, 15]. Of note, although vaccine type did not influence seroprevalence in our study, none of the subjects received mRNA vaccines

We found that a longer interval between the two doses of vaccines was associated with a lower seroprevalence of anti-SARS-CoV-2 IgG antibodies. On the contrary, it has been demonstrated that a three-month interval between the primary vaccine dose and the booster might result in a better immune response compared to a short dose interval, when vector vaccines were concerned [16]

In the current study, neither univariable nor multivariable binary logistic regression analysis showed an association between BMI and seroprevalence of anti-SARS-CoV-2 IgG antibodies after the second dose of vaccination. Obesity can negatively affect the immune system, and vaccine uptake may differ based on BMI. However, in line with our findings, the current COVID-19 trials have shown no difference between groups with normal and obese BMIs in terms of vaccine efficacy [17]. Similarly, no association between BMI and serological response has been reported in cohorts and cross-sectional studies [12, 18, 19]. Contrarily, Pellini et al. have reported that immunogenicity of SARS-CoV-2 vaccine may be impaired by obesity [20]. Consequently, it is necessary to conduct further studies to better understand whether the long-term effectiveness of COVID-19 vaccination depends on individuals’ BMI.

Understanding the immunological reaction that generates a protective immunization to SARS-CoV-2 is crucial [21]. In comparison to the membrane, envelope, and nucleocapsid proteins, antibody responses to the spike protein are considered to be the predominant focus of neutralizing activity during viral infection [22, 23]. However, it is important to note that only a proportion of anti-SARS-CoV-2 spike protein IgG antibodies have neutralizing capacity, and no neutralization assays were performed in the current study. Therefore, the seroprevalence of anti-SARS-CoV-2 spike protein IgG antibodies may not accurately reflect the neutralizing effects of vaccines. It has been demonstrated that declining levels of neutralizing antibodies are associated with an increased risk of symptomatic infection, although the relationship is less clear for severe infections [24]

The current investigation had some limitations. The association between seroprevalence and the number of vaccine doses could not be evaluated since only those staff members who received the second vaccine dose were tested again for anti-SARS-CoV-2 IgG. Moreover, although we took prior COVID-19 infection into account, it is not clear how long ago the infection occurred. This is important because IgG titer attenuates over time. Also, we did not assess neutralizing antibodies and cell-mediated immune responses

## Conclusions

The seroprevalence of anti-SARS-CoV-2 IgG was around 50% in our children’s hospital staff members and HCWs after the second vaccination with vector or inactive virus vaccines. Contrary to previous studies, higher age and lower between-dose intervals led to increased seroprevalence. Despite the results of several studies in this regard, there is a need for further investigations to determine the seroprevalence of anti-SARS-CoV-2 IgG, especially in Iran, because the employed vaccine types are quite different from many other countries

## Data Availability

The datasets used and/or analyzed during the current study are available from the corresponding author on reasonable request.
